# Ankle Joint Biomechanics in Recreational Runners with Resolved and Incident Plantar Fasciitis: A One‐Year Prospective 4HAIE Cohort Study

**DOI:** 10.1111/sms.70281

**Published:** 2026-04-13

**Authors:** Jan Plesek, Joseph Hamill, Pavel Brtva, Jiri Skypala, Jan Urbaczka, David Zahradnik, Julia Freedman Silvernail, Lukas Cipryan, Milos Golian, Jan Sustek, Daniel Jandacka

**Affiliations:** ^1^ Department of Human Movement Studies, Human Motion Diagnostic Centre University of Ostrava Ostrava Czech Republic; ^2^ Department of Kinesiology, Biomechanics and Motor Control Laboratories University of Massachusetts Amherst Massachusetts USA; ^3^ Sport Sciences–Biomedical Department, Faculty of Physical Education and Sport Charles University Prague Czech Republic; ^4^ Department of Kinesiology and Nutrition Sciences University of Nevada Las Vegas Las Vegas Nevada USA; ^5^ Institute for Research and Applications of Fuzzy Modeling University of Ostrava, CE IT4Innovations Ostrava Czech Republic

**Keywords:** foot pain, heel pain, kinematics, kinetics, plantar fasciopathy, risk factors

## Abstract

The aim of this study was to compare running biomechanics between retrospective and prospective cases of plantar fasciitis, and healthy matched controls. A further analysis from the 4HAIE cohort, the current study included four groups: 10 prospectively newly injured runners with plantar fasciitis during the 1‐year follow‐up (PPF), 14 retrospectively injured runners with no report of any prospective lower limb injury (RPF; resolved plantar fasciitis), and two groups of 24 matched healthy controls (10 CON1 and 14 CON2; matching criteria: sex, age, weakly running distance, BMI, height, mass, and body fat). All participants completed eight overground running trials, during which three‐dimensional kinematic and kinetic data were collected. Key variables included vertical component of ground reaction force (VGRF) and ankle joint angle waveforms. Statistical parametric mapping using paired and independent *t*‐tests was performed to identify differences between groups. In addition, baseline MRI of plantar fascia and inflammatory markers were evaluated as control variables. The most notable findings were observed in the frontal plane: the PPF group showed significantly greater ankle eversion compared to CON1 (between 10%–100% stance, *p <* 0.001) and RPF (0%–100% stance, *p <* 0.001). In addition, both PPF and RPF displayed greater VGRF than their controls (25%–50% stance, *p <* 0.001). These findings offer new insights into plantar fasciitis‐related running biomechanics. Prevention and management may benefit from altered technique and motion‐control/stability footwear or insoles, particularly in runners with a history of plantar fasciitis and in uninjured runners exhibiting elevated ankle eversion and increased VGRF near peak eversion.

## 1 Introduction

Plantar fasciitis is among the most common running‐related injuries [1]. However, it does not always prompt runners to interrupt their daily training routines or seek professional medical attention [2]. Despite this, it is evident that the condition causes a certain degree of discomfort, often leading to a reduction in training frequency or volume [3]. While numerous publications have addressed treatment strategies [4, 5, 6], relatively few prospective studies have explored the risk factors that may contribute to the development of plantar fasciitis in runners [7, 8].

It is well established that running injuries are multifactorial in nature (running technique, training errors, sex, footwear, surface, running experience etc.) as is typical for overuse injuries [9]. Among the most significant risk factors are a history of previous injury, high weekly running volume and running biomechanics [8, 9, 10]. In addition to these external and biomechanical aspects, tissue‐level processes such as extracellular matrix remodeling and low‐grade chronic inflammation may play important roles in the onset and progression of plantar fasciitis. Chronic mechanical stress may lead to inflammation, degenerative changes and altered mechanical properties of the fascia, which further increase pain and functional limitations [11]. Pro‐inflammatory cytokines, such as interleukin‐1β (IL‐1β) and tumor necrosis factor‐α (TNF‐α), may play a role in the early stages of tissue degeneration and pain sensitization, even before clinical symptoms appear [11, 12, 13, 14]. Magnetic resonance imaging (MRI) is useful for detecting subclinical changes in the plantar fascia, including thickening, oedema, and altered signal intensity [15, 16], which may indicate early stages of the condition. These findings support the idea that some runners may exhibit tissue‐level pathology without obvious symptoms [17], potentially influencing their biomechanics.

In terms of running biomechanics, previous research primarily using retrospective study designs, have investigated whether running technique and associated vertical ground reaction forces (VGRF) may play a role in plantar fasciitis [18, 19, 20, 21]. These studies typically involved runners who ran at least 20 km per week. Based on their findings, increased loading rates following initial foot‐ground contact were frequently identified as factors associated with plantar fasciitis, although their causal role remains uncertain due to the retrospective nature of the studies [18, 20, 21]. However, these proposed risk factors were not supported by our recent prospective cohort study that included wide range of runners from 18–65 years who regularly have ran from 6 to more than 41 km/week [8]. Additionally, the abovementioned study identified peak rearfoot eversion during stance as a potential biomechanical risk factor for plantar fasciitis (an increase of one degree to the eversion indicates a 19% higher risk) while greater peak ankle abduction (toe‐out) was displayed in the healthy controls. Moreover, recent cross‐sectional study by Kongtong & Khongprasert (2025) [22] showed also greater peak rearfoot eversion in runners with plantar fasciitis compared to their controls. Nevertheless, these studies, like many others, relied on/investigated discrete variables such as maxima, minima, range of motion or values at specific time points (e.g., initial contact), without considering the continuous nature of joint motion—particularly at the ankle—throughout the stance phase. A recent prospective study by Plesek et al. (2025) [8] did not include individuals with a prior history of plantar fasciitis in the biomechanical analysis. On the contrary, a retrospective cross‐sectional study by Wiegand et al. (2022) [19] compared runners with acute plantar fasciitis, runners with a history of plantar fasciitis but no current symptoms (resolved), and healthy controls; however, this study lacked prospectively injured individuals with plantar fasciitis.

From this perspective, it would be valuable to examine runners who were: (1) asymptomatic at the start of the study but had a history of plantar fasciitis and who remained injury‐free during the 1‐year follow‐up; (2) runners who newly developed plantar fasciitis prospectively during the follow‐up; and (3) healthy controls with no plantar fasciitis history before or during follow‐up (those who remained injury‐free throughout). The inclusion of two matched‐pair control groups allowed us to compare one matched group with the prospective, and another with the retrospective group, which methodologically provides less biased comparisons (Table 1). In general, this approach may offer deeper insight into the biomechanical characteristics of running technique across different stages of the injury, particularly when combined with waveform analysis which preserves the natural structure of the data and avoids issues that can arise from discretization (e.g., initial contact, minimum, maximum etc.) [23]. This injury‐stage‐specific waveform analysis could help distinguish between risk‐related biomechanical characteristics and compensatory adaptations, offering valuable insights for prevention and rehabilitation strategies.

**TABLE 1:** Descriptive characteristics of the groups. Matching criterion: Sex, age, weekly running distance, BMI, height, mass, body fat (Paired t‐test).

	PPF	CON1	*p*	*d*	RPF	CON2	*p*	*d*
*N*	10	10			14	14		
Key Characteristics (Matching‐Criteria)								
Sex (F/M; *N* (%))	5/5 (50%/50%)	5/5 (50%/50%)			7/7 (50%/50%)	7/7 (50%/50%)		
Age (year)	37.5 ± 11.2	37.9 ± 11.2	0.343	0.04	38.4 ± 8.2	38.6 ± 8.0	0.435	0.02
Running distance—retrospective questionnaire (km/week)	29.1 ± 9.7	29.4 ± 10.1	0.745	0.03	31.2 ± 20.3	31.9 ± 18.9	0.138	0.05
BMI	22.3 ± 2.4	22.1 ± 1.2	0.703	0.11	22.8 ± 1.8	22.9 ± 1.5	0.818	0.06
Height (cm)	178.4 ± 7.9	177.5 ± 8.8	0.545	0.11	174.3 ± 11.6	174.9 ± 8.4	0.774	0.06
Mass (kg)	71.3 ± 10.8	69.9 ± 10.0	0.587	0.13	69.7 ± 12.8	70.3 ± 9.3	0.831	0.05
Body Fat (%)	26.2 ± 2.4	25.9 ± 3.9	0.815	0.06	27.0 ± 5.7	27.1 ± 6.1	0.867	0.03

*Note:* Data are presented as mean and SD. PPF cases—participants who had no history of plantar fasciitis before baseline measurement and suffered new plantar fasciitis during one‐year follow‐up. CON1—matched‐pair controls for PPF. RPF cases—participants who reported plantar fasciitis before baseline measurement and consequently did not suffer any lower limb injury during one‐year follow‐up. CON2—matched‐pair controls for RPF.

Therefore, the aim of this study was to compare profiles of the vertical ground reaction force (VGRF) component and ankle joint kinematics during the stance phase of running between four groups: prospective onset of new cases of plantar fasciitis in runners (PPF), runners with “resolved” cases of plantar fasciitis (RPF), and two groups of matched healthy controls (by sex, age, running volume, BMI, shoes) using statistical parametric mapping (SPM) for waveform analysis. We hypothesized that we would observe no differences in VGRF between the groups [8, 19]. In addition, we hypothesized that the PPF group would exhibit greater ankle eversion and lower abduction during the stance phase compared to both the RPF and the matched healthy controls (CON1) [8, 22].

## 2 Methods

### 2.1 Study Design and Study Sample Size

This study is the further analysis of the 1‐year prospective, multidisciplinary, 4HAIE cohort study (*N* = 1315) on biomechanical risk factors for plantar fasciitis [8, 24]. The current study focused more specifically on biomechanical risk factors using waveform analysis and included participants who suffered plantar fasciitis on the right foot. This selection was made to ensure higher internal validity of the data, as the overground running task was designed to assess the biomechanics of the right lower limb.

At baseline, 37 participants retrospectively reported a history of plantar fasciitis (from *N* = 1315). Seven participants were excluded: two dropped out during the 1‐year follow‐up, and five others reported a history of plantar fasciitis both retrospectively and concurrently, along with a self‐reported injury similar to plantar fasciitis also reported prospectively (excluded from the analysis).

Among the remaining 30 participants with resolved plantar fasciitis, ten (33%) reported an injury on the left foot (excluded), seven (23%) had a history of plantar fasciitis on the right foot (included), another seven (23%) reported a history on both sides (included), and six (20%) did not report any specific side (missing data; excluded). Hence, 14 runners with resolved right‐foot plantar fasciitis were included in this study.

Additionally, most of the new prospective cases of plantar fasciitis in runners (*N* = 14) were reported on the right foot (*N* = 10; 71%; included), while the remaining cases were on the left foot (*N* = 4; 29%; excluded). Thus, 10 new cases with right‐foot plantar fasciitis were included in this study.

For the purposes of this study, a subsample of 48 runners was included and divided into four groups according to their plantar fasciitis injury status: prospective new plantar fasciitis (PPF); healthy controls to PPF (CON1); resolved plantar fasciitis (RPF); healthy controls to RPF (CON2). Sample size estimation—sensitivity for paired *t*‐tests (PPF‐CON1: 20 participants; and RPF‐CON2: 28 participants; with the statistical power 80%; alpha = 0.05) indicated that the sample was sufficient to detect statistically significant differences on the level d = 0.66 and d = 0.55, respectively. The 4HAIE study was approved by the Ethics and Research Committee of the University of Ostrava (OU‐87674190‐2018) and was conducted in accordance with the principles of the Declaration of Helsinki. All participants signed an informed consent before the data collection.

Independent variable (both retrospective and prospective data): injury status (PPF, RPF, CON1, and CON2).

Main dependent variables (baseline—lab data): profiles of VGRF, ankle angle in sagittal (dorsiflexion/plantar flexion), frontal (inversion/eversion), and transversal plane (adduction/abduction; toe‐in/toe‐out) during stance phase of running.

Main control variables (baseline—lab data): sex, age, weekly running distance (retrospective questionnaire ACLS), BMI, height, mass, body fat.

Additional control variables: training characteristics (prospective—follow up data: e.g. average elevation gained per week during running episodes, average number of running episodes per week and average weekly running distance etc.); VO_2_max, strike index, cadence, MRI plantar fascia evaluation, blood inflammatory and anti‐inflammatory biomarkers (baseline—lab data).

### 2.2 Participants

In the 4HAIE study, a runner was defined as an individual who regularly ran at least 10 km per week for a minimum of 6 weeks (or at least 6 km per week for individuals older than 60 years) and planned to continue running for another 12 months. All runners in the current study (*N* = 48) regularly ran at least 10 km per week. We included 10 runners with no history of plantar fasciitis before the baseline who reported a new condition of plantar fasciitis during 1 year follow‐up (PPF) and 10 non‐injured healthy controls (CON1) who were match‐paired to the PPF group (Table 1) [8]. In addition, we included 14 runners with a history of plantar fasciitis prior to baseline who remained free of lower‐limb injuries during the 1‐year follow‐up (RPF), along with 14 non‐injured healthy controls (CON2) matched to the RPF group. Prospectively injured runners (PPF) were assessed and confirmed by medical professionals after an injury report (via the 4HAIE application (HealthReact) or phone call). For the purposes of this study, a previous history of plantar fasciitis was classified as resolved if participants had been asymptomatic and fully participating in their normal running routine for at least 6 weeks prior to the baseline and remained injury‐free throughout the 12‐month follow‐up period (consistent with IOC consensus definitions of full recovery) [17, 19, 25].

All runners wore neutral standard running (cushioned) shoes during their runs. In 63% of the participants, runners ran on a hard surface such as asphalt/hard‐surfaced road (PPF = 90%; CON1 = 70%; RPF = 50%; CON2 = 50%) with 37% of runners running on unpaved roads or natural surfaces (PPF = 10%; CON1 = 30%; RPF = 50%; CON2 = 50%). Only 29% of runners ran in an urban environment (equally distributed among the groups).

Ninety‐six percent of runners had been running regularly for more than 1 year before the baseline. Most of them had been running for 2–5 years (38%), followed by 5–8 years (27%) and more than 8 years (17%). In the PPF group, 10% had been running for less than half a year; 10% for 1–2 years; 50% for 2–5 years; 20% for 5–8 years; and 10% for more than 8 years. In CON1, 10% had been running for 1–2 years; 60% for 2–5 years; and 30% for 5–8 years. In RPF, 14% had been running for 1–2 years; 21% for 2–5 years; 29% for 5–8 years; and 36% for more than 8 years.

Seventy percent of PPF runners reported spending their work time in sedentary or standing positions, while RPF runners and CON2 reported less sedentary or standing activity—64% and 57%, respectively. In contrast, CON1 worked sedentary only in 40%.

### 2.3 Protocol and Setting

Baseline assessments were conducted over two consecutive days. On the first day at 6:00 p.m., participants reported to the Human Motion Diagnostic Centre, where they underwent a series of initial evaluations. These included completing physical activity questionnaires, anthropometric measurements (body height and mass), and assessments of resting blood pressure (data not shown), cardiorespiratory fitness, resting spirometry (data not shown), and a graded exercise test to exhaustion (VO_2_peak). Detailed procedures are outlined in the methodological papers [26, 27].

The Aerobics Center Longitudinal Study survey (ACLS) [28] and the Running Status and History questionnaire (RUNHIS) [2], were used to retrospectively describe runners characteristics at the baseline (weekly running mileage, running shoes, running surface, running environment, running experience etc.) For instance, data from the ACLS survey contained questions: “During the last two months, which of the following moderate or vigorous activities have you performed regularly? – Jogging or running?” (Yes/No), “How many sessions per week?”, and “How many kilometers per session?” Weekly running distance was then calculated by multiplying the number of sessions by the distance per session.

On the second day, fasting blood samples (30 mL) were collected from the antecubital vein in the morning hours (06:30–07:00) within the physiology laboratory and followed by anthropometric body composition assessment (body dimensions and composition). Magnetic resonance imaging (MRI; 1.5 T Magnetom Sempra scanner; Siemens, Erlangen, Germany) was performed followed by anthropological assessments. Ankle joint scans were conducted using a 16‐channel head coil modified for foot‐ankle (including plantar fascia and Achilles' tendon). The head coil was equipped with a custom‐designed device to secure the leg at a 90‐degree angle. Participants lay in a supine position with their ankle immobilized and they were instructed to remain completely still throughout the scan. The total acquisition time was approximately 20 min. The MRI measurements were focused on the region of the ankle joint, the Achilles tendon, and the calcaneus, including the plantar fascia and the calcaneal tuberosity. Protocol included five sequences: sagittal T1‐weighted spin echo (SAG T1 SE), transverse T2‐weighted turbo spin echo (TRA T2 TSE), coronal T1‐weighted turbo spin echo (COR T1 TSE), sagittal and transverse proton density–weighted turbo spin echo with fat suppression (SAG/TRA PD TSE FS), and sagittal T2* mapping (multi‐echo GRE). The selection was tailored to evaluate the plantar fascia (thickness and morphology) and to detect fasciitis on fluid‐sensitive images (hyperintense fascial signal with peri‐fascial/bone‐marrow edema).

Finally, participants completed an overground running protocol at a self‐selected speed corresponding to their typical training pace. Participants first reported their typical training pace (min/km), which we converted to a target speed (m/s) to guide self‐selection. The speed was the objectively measured average from photocell timing gates. Two timing gates were positioned 3 m apart straddling the force plate (at the midpoint of a 17‐m runway); the measured speed represented the average over this 3‐m segment centered on the force plate (i.e., reflecting speed at the force platform), not the average across the full 17 m. Participants then ran back and forth for 2 min; during the final 30 s, four runs (not captured by the motion capture system) were measured by photocells timing gates, and their mean defined each participant's self‐selected speed. The overground running task consisted of eight successful trials performed at the self‐selected speed within ±5% and recorded by the 3D motion capture system. Each recorded trial was initiated from rest (standing start) on a verbal instruction; the starting point was at least 7 m before the force plate. After each discontinued running trial, participants walked back to the start position. A trial was considered valid if the participant stepped onto the force plate, landing approximately at its center, with the entire right foot. Recording continued until eight valid trials were obtained; invalid trials (e.g., off‐target foot strikes or speed outside ±5%) were repeated. The overground running task for a single participant usually did not exceed 15 min. Across participants, the total number of running trials typically ranged from 10 to 20.

### 2.4 Laboratory Set‐Up, Instrumentation and Marker Placement

**FIGURE 1:**
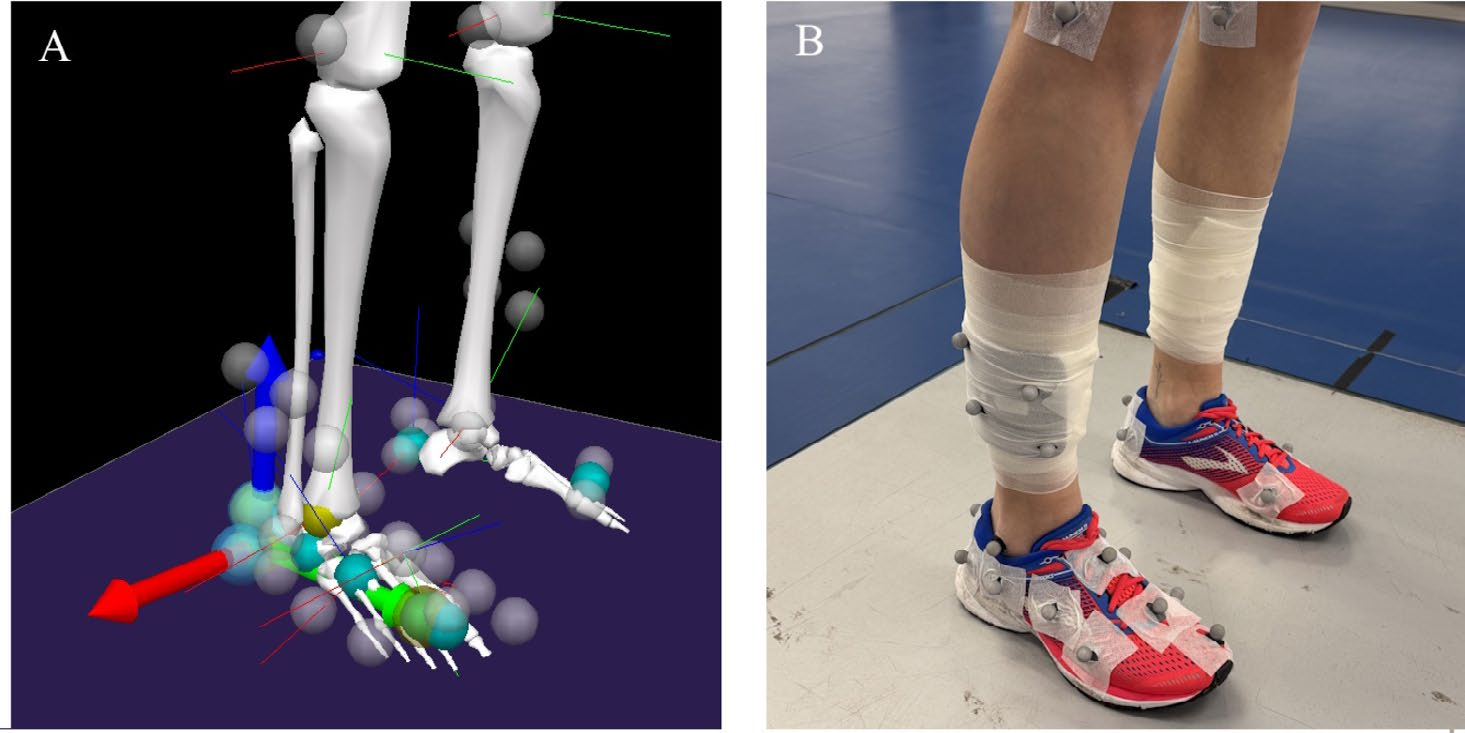
Marker set up. Virtual foot model: Axis X (red, medio‐lateral); Y (green, anterior–posterior), Z (blue, vertical) (A). Marker set up and running shoes (B).

Kinematic and kinetic data were collected by using a 10‐camera 3D motion capture system (Oqus, Qualysis Inc., Gothenburg, Sweden) sampling at 240 Hz and recorded synchronously with ground reaction forces from the force platform sampling at 2160 Hz (90 × 90 cm 9287CCAQ02 Kistler, Winterthur, Switzerland). Four reflective tracking markers were attached bilaterally to the pelvis at the posterior and anterior superior iliac spines. Additionally, ten calibration markers were placed on both sides of the body at the medial and lateral malleoli, medial and lateral femoral condyles, and the greater trochanter of the femur. Four lightweight rigid plates, each with four markers, were mounted on the thigh and shank. Thirteen markers were positioned on the right running shoe following the multi‐segmental Rizzoli foot model (Figure 1A,B). However, for the purposes of data analysis in this study, only a triad of tracking markers on intact shoe (over the heel) and two markers on the first and fifth metatarsal heads were used as this simplified setup provided greater reliability and objectivity compared to the full multi‐segmental model [29]. Before the biomechanical measurement, a standing calibration trial was recorded. All participants wore standard laboratory neutral running shoes (Brooks Launch 5; Brooks Sport Inc., Seattle, WA) (Figure 1B).

Anthropometric data (body height and mass) of all participants were measured by a stadiometer (In Body 370, Biospace, South Korea) and body composition analyzer (Inbody 770, Biospace, South Korea), respectively. Body composition parameters of fat and fat‐free mass (lean mass) were measured by dual‐energy X‐ray absorptiometry (DEXA; Hologic Discovery A, USA). Magnetic resonance data were acquired at a 1.5 T Magnetom Sempra scanner (Siemens, Erlangen, Germany).

Prospectively monitored physical activity (during 1 year follow‐up) was monitored via Fitbit Charge 3 or 4 monitors (Fitbit, San Francisco, USA).

### 2.5 One‐Year Follow Up and Injury Reporting

Participants wore physical activity monitors continuously for 1 year. In terms of reporting running related injuries, we used three methods/approaches: (1) a self‐initiated injury report by participant via a mobile app (4HAIE app); (2) a weekly injury survey administered every Sunday between 4:00 and 8:00 p.m.; or (3) a triggered survey if a decline in physical activity was detected by the Fitbit device [26]. In cases where injury reports were unclear, a physiotherapist from the 4HAIE team contacted the participant to verify the injury and confirm whether medical assessment was sought and a medical diagnosis was made. Only participants with plantar fasciitis (PPF) confirmed by a medical doctor or physiotherapist were included in the study. Participants were provided with the opportunity to consult an orthopedic specialist affiliated with the study. However, due to the broad geographic distribution of the 4HAIE cohort, they were also permitted to seek consultation from their usual healthcare provider or orthopaedist. Additionally, participants were asked whether their plantar fasciitis was related to sports activity, and if so, whether it was specifically caused by running.

### 2.6 Data Processing

#### 2.6.1 Biomechanics

All biomechanical data were processed using Qualisys Track Manager (Qualisys, Sweden). Further data processing was performed in Visual 3D software (C‐motion, USA). A low–pass Butterworth filter with a cut‐off frequency of 12 Hz and 50 Hz was applied for the motion capture data and for force data, respectively [24]. Three‐dimensional (3‐D) knee and ankle joint angles were calculated using an X‐y‐z Cardan rotation sequence. Ankle angles were determined as the relative position of the right foot to the right shank. A virtual foot model was used for the description of ankle angle motion in order to better clinical interpretation. Rotations around the axis and description of the ankle movement followed the right thumb rule. Specifically, rotations around the *X* axis (Figure 1; red) describe a movement in the sagittal plane of the ankle (dorsiflexion +/plantarflexion‐), rotations around *Y* (green) correspond to the frontal plane (inversion +/eversion‐), and rotation around the *Z* (blue) represented movement in the transversal plane of the ankle (adduction; toe‐in +/abduction; toe‐out‐). Signals of VGRF and ankle angles during the stance phase of the right lower limb were normalized into 101 points (0%–100% stance) for following statistical parametric mapping analysis [23]. The strike index was estimated based on the location of the centre of pressure at the initial foot‐ground contact and reported as a relative length (%) from the posterior calcaneus [30].

#### 2.6.2 Magnetic Resonance Imaging

Additional (ad‐hoc; in September 2025) plantar fascia MRI data of 46 participants (two participants had missing MRI data; Table S1) were evaluated by an experienced radiologist with over 15 years of clinical experience in musculoskeletal imaging. Participant group assignment was blinded to the radiologist. Plantar fascia was evaluated using sagittal T1‐weighted spin‐echo (thickness), coronal T1‐weighted turbo spin‐echo (thickness), and sagittal proton‐density–weighted turbo spin‐echo sequences with fat suppression (thickness, intensity of the signal, and oedema). The radiologist assessed thickness of the plantar fascia (thickness < 4 mm or thickness ≥ 4 mm), signal intensity (low, intermediate, heterogeneous hyperintensity), oedema in the adjacent soft tissues or calcaneal bone marrow oedema (yes/no), and possible partial tears of plantar fascia (yes/no) [15, 16]. Thickness was measured on all three aforementioned sequences, and the mean of the three measurements was calculated. This mean value was subsequently categorized into two groups (< 4 mm or ≥ 4 mm). The plantar fascia thickness was measured at the point of maximal thickness of the central component of the plantar aponeurosis, located within 1 cm of the calcaneal insertion (medial tubercle of the calcaneus). Measurements were performed using orthogonal line placement perpendicular to the long axis of the fascial fibers [31]. Tissue borders were delineated based on characteristic MRI signal intensity patterns. The plantar fascia was identified as a well‐defined hypointense linear structure, clearly distinguishable from the adjacent plantar fat pad (high signal intensity on T1‐weighted images) and intrinsic plantar muscles (intermediate signal intensity).

We used grading of plantar fascia: grade 0—healthy plantar fascia, thickness < 4 mm with low signal intensity, no oedema and no tears; grade 1—thickness ≥ 4 mm with low signal intensity, no oedema and no tears; grade 2—thickness ≥ 4 mm with intermediate intensity of the signal without oedema and no tears; grade 3—thickness ≥ 4 mm with heterogeneous hyperintensity of the signal and extensive oedema, or possible tears of plantar fascia. Representative examples of each grade are shown in Supplemental Figure 1. Consequently, we dichotomized MRI grading of the plantar fascia into two categories: intact or non‐pathological fascia (grades 0–1, representing normal to mildly altered tissue), and pathological fascia (grades 2–3, representing moderately to severely altered tissue).

For reliability assessment, the radiologist re‐evaluated MRI scans of 12 randomly selected participants representing all grading categories (0–3) to determine intra‐reader consistency. Test–retest agreement between the two time points (September 2025 and January 2026) for the ordinal grading showed excellent reliability (quadratically weighted κ = 0.94, 95% CI 0.78–1.00). As a sensitivity analysis, we additionally calculated ICC (3,1; two‐way mixed model, consistency), which was also excellent (ICC = 0.947, 95% CI 0.828–0.985, *p* < 0.001). Overall, the radiologist's assessments demonstrated excellent intra‐reader reliability.

#### 2.6.3 Biochemical Blood Analysis

The biochemical blood analysis was provided by an external specialized and certified biochemical laboratory with long‐term experience in clinical analysis and research projects. For the purposes of this study, we analyzed these inflammatory biomarkers: interleukin‐1β (IL‐1β), interleukin‐1 receptor antagonist (IL‐1RA), interleukin‐6 (IL‐6), interleukin‐10 (IL‐10), tumor necrosis factor‐α (TNF‐α), high‐sensitive C reactive protein (CRP) [14, 17]. It was shown that pro‐inflammatory and anti‐inflammatory cytokines may play an important role in chronic diseases/injuries or potentially be used as early predictors for running‐related injuries through initiation of healing or facilitating tissue repair [14].

#### 2.6.4 Physical Activity Data (Fitbit)

Weekly running distance and average elevation gained were obtained using a custom script in Python from the Fitbit Charge 3/4 fitness tracker based on minute‐to‐minute accelerometer and barometer data. These data were calculated as weekly average values from active running weeks. The definition of an active running week followed the criteria outlined in the protocol paper [24]. Specifically, participants were required to report a minimum of 75 min or 6 km of running during that week. We calculated running‐related characteristics, including average weekly running distance (km/week), average number of running episodes per week (N/week), average weekly total duration of running episodes (min/week), average duration of a single running episode (min), average speed during running episodes (km/h), and average elevation gained per week (m/week) during running episodes. All metrics were computed as averages across complete active running weeks (Monday–Sunday). Partial weeks at the beginning or end of follow‐up were excluded. For prospectively injured runners (PPF), the averaging period included only the weeks preceding the first prospectively reported plantar fasciitis.

### 2.7 Statistical Methods

A two‐tailed paired *t*‐test was performed for normally distributed data or a Wilcoxon signed rank test for non‐normally distributed data to compare anthropometric, running characteristics, and blood cytokines levels of participants between paired groups (Table 1). A two‐tailed independent *t*‐test or Mann Whitney U test was used for PPF‐RPF and CON1‐CON2 differences. Normality of the data was assessed by Shapiro–Wilk test. Statistical level of significance was set at *p* = 0.05.

A Wilcoxon signed rank test (PPF‐CON1 and RPF‐CON2) and Mann Whitney U test (PPF‐RPF and CON1‐CON2) were used to compare ordinal data of MRI grading of plantar fascia at the baseline. Statistical level of significance was also set at *p* = 0.05.

The 3‐D ankle motion curves (X‐Y‐Z) and VGRF component were analyzed in Matlab (MATLAB R2017a, Mathworks Inc., Natick, MA, USA) and an open‐source SPM software package was used to conduct the statistical analysis of the ankle angles and VGRF during the stance phase [23]. Statistical parametric mapping allows researchers to perform statistical analyses directly on smooth, continuous biomechanical data such as movement patterns over time without needing to break them into arbitrary segments or discrete events (initial contact, maximum, minimum, etc.). This approach helps preserve the natural structure of the data and avoids issues that can arise from discretization [23]. A two‐tailed paired *t*‐test was used for related samples paired groups (PPF‐CON1; 80–80 running trials kinematics and VGRF data) and (RPF‐CON2; 102–102 trials for kinematics; 108–108 trials for VGRF) and a two‐tailed independent *t*‐test was performed for the PPF‐RPF comparison (also additionally for control groups comparison (CON1‐CON2)). Numbers and distribution of missing data can be seen in the Table S1. Statistical level of significance was also set at *p* = 0.05.

Biological relevance (practical significance) was expressed as effect size assessed by Cohen's *d* (small < 0.50; medium 0.50 < 0.80; large ≥ 0.80) and by Rank‐biserial correlation *r* (small 0.10 < 0.30; medium 0.30 < 0.50; large ≥ 0.50) [32, 33].

## 3 Results

### 3.1 Anthropometrics and Running Characteristics

No differences were found between paired groups (PPF‐CON1 and RPF‐CON2) in sex, age, BMI, height, mass, percentage of fat, weekly running volume (ACLS), prospectively measured average weekly running distance, average total duration of running activity per week, average number of running episodes per week, average weekly, average duration of one running episode, average running speed in running episodes, average elevation gained during running per week, VO_2_max, strike index, cadence (*p* > 0.05) (Table 1 and Table 2).

**TABLE 2:** Running characteristics, MRI of plantar fascia, and cytokines (Paired *t*‐test or Wilcoxon signed rank test).

	PPF	CON1	*p* (*d* or *r*)	RPF	CON2	*p* (*d* or *r*)
Running Characteristics						
**Running Physical Activity—**prospectively measured by Fitbit during follow‐up	*N* = 9	*N* = 9		*N* = 13	*N* = 13	
*Average running distance per week (km/week)*	28.9 (14.5–36.2)—up to PPF injury	19.0 (14.9–21.4)	0.173 (0.45)	19.4 (17.4–34.1)	20.9 (14.2–28.4)	0.311 (0.28)
*Average duration of running activity per week (min/week)*	168 (80–231)—up to PPF injury	119 (82–138)	0.110 **(0.53)**	124 (101–204)	115 (85–162)	0.345 (0.26)
*Average number of running episodes per week (N/week)*	3.22 (2.80–4.82)—up to PPF injury	3.45 (2.86–4.10)	0.767 (0.10)	4.00 (3.10–5.18)	3.29 (2.53–4.73)	0.173 (0.38)
Average duration of single running episode (min)	41.4 ± 15.4—up to PPF injury	31.2 ± 7.3	0.065 (0.78)	33.7 ± 11.0	36.7 ± 15.4	0.525 (0.22)
Average running speed in running episodes (km/h)	9.99 ± 0.78—up to PPF injury	9.71 ± 0.89	0.408 (0.34)	9.7 ± 0.8	9.8 ± 0.9	0.823 (0.07)
*Average elevation gained per week (m/week)*	175.6 (72.1–290.5)—up to PFF injury	154.9 (115.0–298.2)	0.953 (0.20)	194.3 (166.0–443.3)	177.5 (94.4–469.4)	0.552
**Aerobic capacity—baseline**	*N* = 10	*N* = 10		*N* = 14	*N* = 14	
VO2Max – lab (ml/min/kg)	48.7 ± 7.9	46.0 ± 6.9	0.073 (0.35)	48.7 ± 7.0	46.0 ± 8.2	0.183 (0.35)
**Running Biomechanical variables—baseline**	*N* = 10	*N* = 10		*N* = 13	*N* = 13	
*Strike index—lab (%)*	10.0 (1.4–52.8)	10.8 (7.2–29.4)	0.721 (0.11)	13.6 (7.8–65.1)	11.2 (6.6–18.0)	0.152 (0.38)
Strike index	24.6 ± 27.5	19.7 ± 20.9		34.6 ± 30.3	15.3 ± 18.3	
Footfall pattern – strike index (RFS/MFS/FFS)	6/4/0	8/1/1		7/4/2	11/1/1	
RFS/NRFS (%)	60%/40%	80%/20%		54%/46%	85%/15%	
Cadence—lab (steps/min)	162.3 ± 9.0	160.8 ± 8.6	0.707 (0.17)	164.4 ± 8.3	162.3 ± 11.4	0.620 (0.21)
**MRI data—baseline**						
**Morphological/structural alteration of plantar fascia**	*N* = 9	*N* = 9		*N* = 13	*N* = 13	
Grade: 0 – normal (*N*/%)	4 (45%)	7 (78%)		8 (62%)	11 (85%)	
Grade: 1—mild (*N*/%)	1 (11%)	0 (0%)		1 (7.5%)	1 (7.5%)	
Grade: 2—moderate (*N*/%)	1 (11%)	1 (11%)		0 (0%)	0 (0%)	
Grade: 3—severe (*N*/%)	3 (34%)	1 (11%)		4 (30.5%)	1 (7.5%)	
	100%	100%		100%	100%	
Plantar fascia (non‐pathological/pathological) (*N*/%)	5/4 56%/44%	7/2 78%/22%		9/4 69.5%/30.5%	12/1 92.5%/7.5%	
*Group score (Median Q1‐Q3)*	1 (0–3)	0 (0–0)	0.157 (0.47)	0 (0–1)	0 (0–0)	0.180 (0.34)
**Blood inflammatory and anti‐inflammatory biomarkers (cytokines)—baseline**	*N* = 8	*N* = 8		*N* = 14	*N* = 14	
*IL‐1β (pg/mL)*	0.45 (0.19–1.39)	0.37 (0.24–1.50)	0.611 (0.18)	0.51 (0.20–1.50)	0.48 (0.12–1.50)	0.944 (0.02)
*IL‐1RA (pg/mL)*	627 (509–857)	423 (286–681)	0.208 (0.45)	352 (289–538)	406 (324–463)	0.964 (0.04)
*IL‐6 (pg/mL)*	0.85 (0.29–1.29)	0.45 (0.12–0.66)	0.091 (**0.60**)	0.07 (0.04–0.30)	0.32 (0.07–0.73)	0.158 (0.38)
*IL‐10 (pg/mL)*	0.15 (0.12–0.15)	0.18 (0.09–0.44)	0.310 (0.36)	0.11 (0.03–0.45)	0.20 (0.12–0.50)	0.330 (0.26)
*TNF‐α (pg/mL)*	2.51 (0.44–3.20)	1.00 (0.43–1.99)	0.036* (0.74)	0.55 (0.19–0.91)	0.92 (0.17–1.70)	0.311 (0.27)
	*N* = 9	*N* = 9		*N* = 14	*N* = 14	
*CRP (mg/L)*	1.65 (0.50–2.40)	0.50 (0.50–0.50)	0.080 **(0.58)**	0.50 (0.50–1.23)	0.50 (0.50–1.45)	0.944 (0.02)

*Note:* Data are presented as mean and SD or *Median and Q1–Q3* or N (%) from complete pairs. Italic represents variables with non‐normal distribution. *Asterisk indicates statistically significant differences. Bold values represent large effect size. PPF cases – participants who had no history of plantar fasciitis before baseline measurement and suffer new plantar fasciitis during one‐year follow‐up. CON1—matched‐pair controls for PPF. RPF cases —participants who reported plantar fasciitis before baseline measurement and consequently did not suffer any lower limb injury during one‐year follow‐up. CON2—matched‐pair controls for RPF.

Independent *t*‐test showed no differences between PPF‐RPF (N: 10/14) and CON1‐CON2 (N: 10/14) in age (small effects; *d* = 0.10 and *d* = 0.07), BMI (small and moderate; *d* = 0.21 and *d* = 0.55), height (small; *d* = 0.40 and *d* = 0.30), mass (small; *d* = 0.13 and *d* = 0.04), percentage of fat (small; *d* = 0.14 and *d* = 0.24), VO_2_max (small; *d* = 0.01 and *d* < 0.01), cadence (small; *d* = 0.25 and *d* = 0.08), average speed during running episodes (N: 9/14; *p* = 0.324; *d* = 0.42; and N: 10/13; *p* = 0.742, *d* = 0.14).

The Mann Whitney U test did not show any differences between PPF‐RPF (*N*: 9/14) and CON1‐CON (*N*: 10/13) with small effects: average weekly running distance (*p* = 0.829; *r* = 0.05; and *p* = 0.648; *r* = 0.10), average number of running episodes per week (*p* = 0.516; *r* = 0.15; and *p* = 0.784; *r* = 0.07), average weekly total duration of running episodes (*p* = 0.829; *r* = 0.05; and *p* = 0.832; *r* = 0.05), average duration of a single running episode (*p* = 0.201; *r* = 0.28; and *p* = 0.410; *r* = 0.18), average elevation gained per week (*p* = 0.277; *r* = 0.24; and *p* = 0.648; *r* = 0.10) during running episodes, and strike index (*N*: 10/14; *p* = 0.285, *r* = 0.23, and *N*: 10/13; *p* = 0.693; *r* = 0.09).

### 3.2 MRI and Cytokines

A Wilcoxon signed rank test showed marginally non‐significant differences were found between PPF and CON1 with medium‐large effect size (*p* = 0.157; *r* = 0.47; *N* = 18) in MRI grading of plantar fascia at the baseline. In addition, there were no differences between RPF and CON2 (*p* = 0.180; *r* = 0.34; *N* = 26) with medium effect size. A Mann Whitney U test showed no statistical differences between PPF‐RPF (*p* = 0.471, *r* = 0.16; *N* = 23) and CON1‐CON2 (*p* = 0.966, *r* = 0.01; *N* = 23) with small effect size. The sample (*N* = 46) did not include any cases of complete plantar fascia tears.

The baseline blood concentration of most cytokines did not differ significantly between paired groups (*p* > 0.05). However, pro‐inflammatory TNF‐α was higher in the PPF group compared to CON1 with large effect size (*p* = 0.036; *r* = 0.74; *N* = 16), while no significant differences were observed between RPF and CON2 (*p* = 0.331; *r* = 0.27; *N* = 28). Additionally, the Mann Whitney test showed significantly higher levels of IL‐1RA (*p* = 0.009; *r* = 0.67; *N* = 22), IL‐6 (*p* = 0.008; *r* = 0.70; *N* = 22), and TNF‐α (*p* = 0.037; *r* = 0.54; *N* = 22) in PPF compared to RPF with large effect sizes.

### 3.3 Biomechanics

**FIGURE 2:**
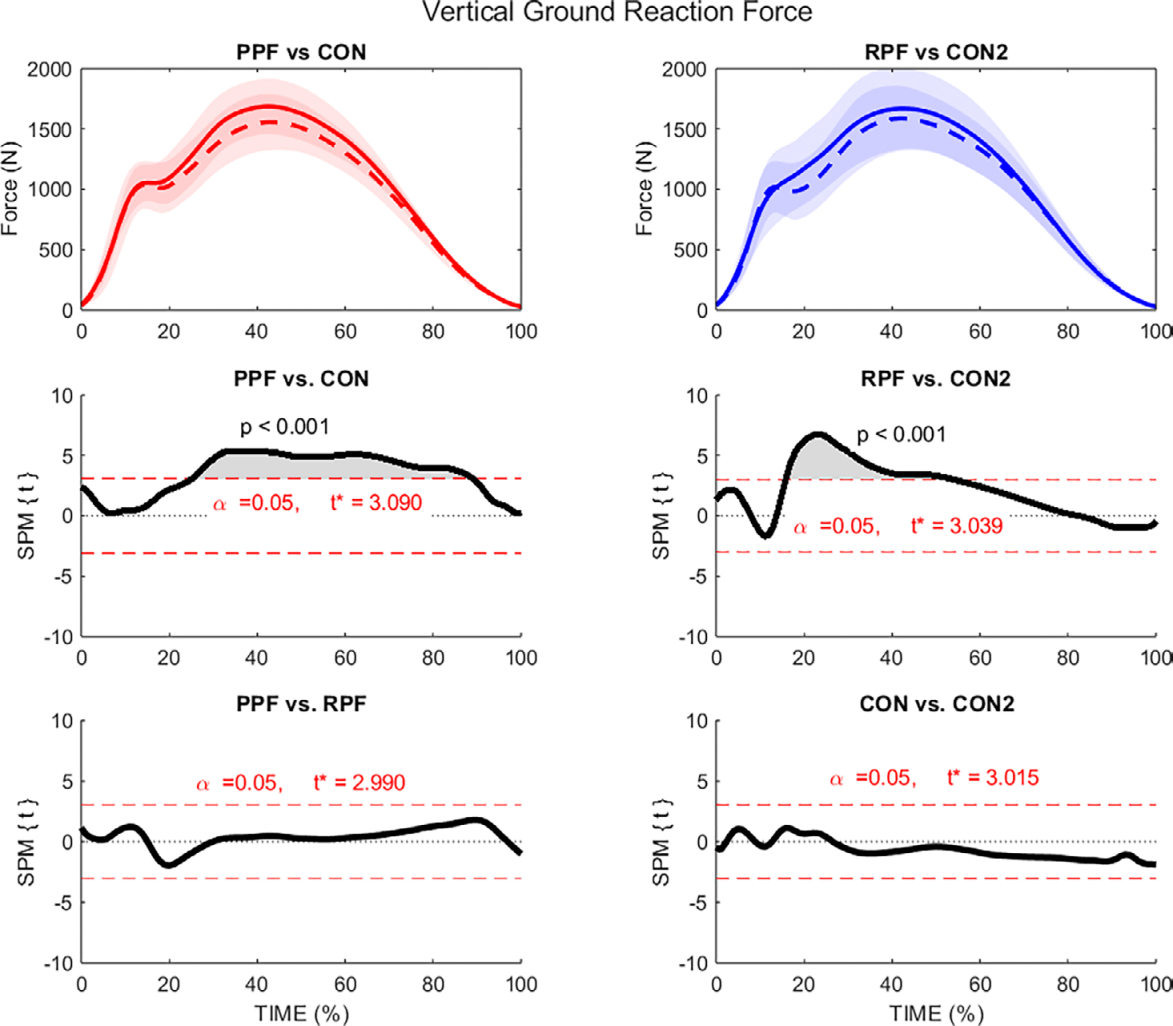
Statistical parametric mapping of vertical ground reaction force during stance phase of running. PPF, red solid line; CON1, red dashed line; RPF, blue solid line; CON2, blue dashed line.

The SPM analysis of the VGRF profiles revealed significant differences between injured groups and controls (Figure 2). The greatest difference in VGRF was found when comparing the PPF group with CON1 (25%–88% stance, *p* < 0.001, average mean difference = 97.9 N, average ES: *d* = 0.60). Additionally, we found greater VGRF in RPF compared to CON2 at approximately 16%–50% of stance (*p* < 0.001, average mean difference = 120.3 N, average ES: *d* = 0.42). No differences were found between PPF and RPF, or between both control groups (*p* > 0.05).

**FIGURE 3:**
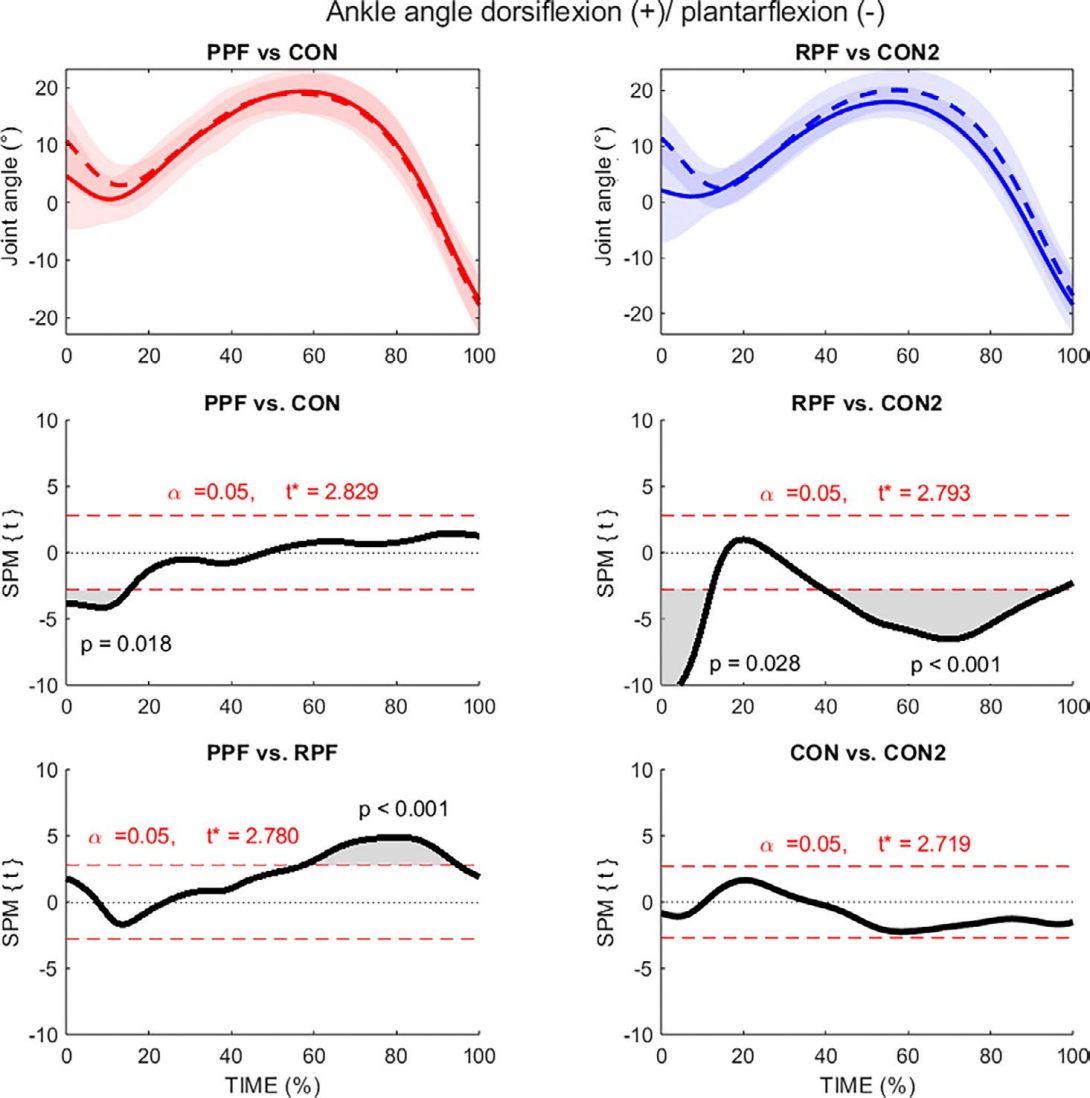
Statistical parametric mapping of ankle joint angle in the sagittal plane (dorsiflexion (+)/plantar flexion (−)) during stance phase of running. PPF, red solid line; CON1, red dashed line; RPF, blue solid line; CON2, blue dashed line.

In terms of sagittal plane, both PPF and RPF displayed a more plantar flexed ankle at initial foot‐ground contact than their control groups (0%–16% stance, *p* = 0.018, average mean difference = 3.8°, average ES: *d* = 0.68; and 0%–13% stance, *p* = 0.028, average mean difference = 5.2°, average ES: *d* = 0.86). The RPF showed greater plantarflexion later in stance compared to PPF (60%–95% stance, *p* < 0.001, average mean difference = 2.9°, average ES: *d* = 0.71) and also compared to its controls CON2 (40%–95% stance; *p* < 0.001, average mean difference = 2.7°, average ES: *d* = 0.63). No differences were found between control groups (Figure 3).

In the frontal plane, the most striking results of this study emerged between the PPF and RPF since the PPF had a greater ankle eversion angle during the entire stance phase than the RPF (0%–100% stance, *p* < 0.001, average mean difference 0–100% stance = 5.4°; average ES: *d* = 0.85).

The PPF group also displayed a significantly greater eversion angle than CON1 (14%–100% stance, *p* < 0.001; average mean difference 14%–100% stance = 2.9°; average ES: *d* = 0.47). The highest effect size between PPF and CON1 occurred at 25% (mean difference = 3.8°; *d* = 0.70).

**FIGURE 4:**
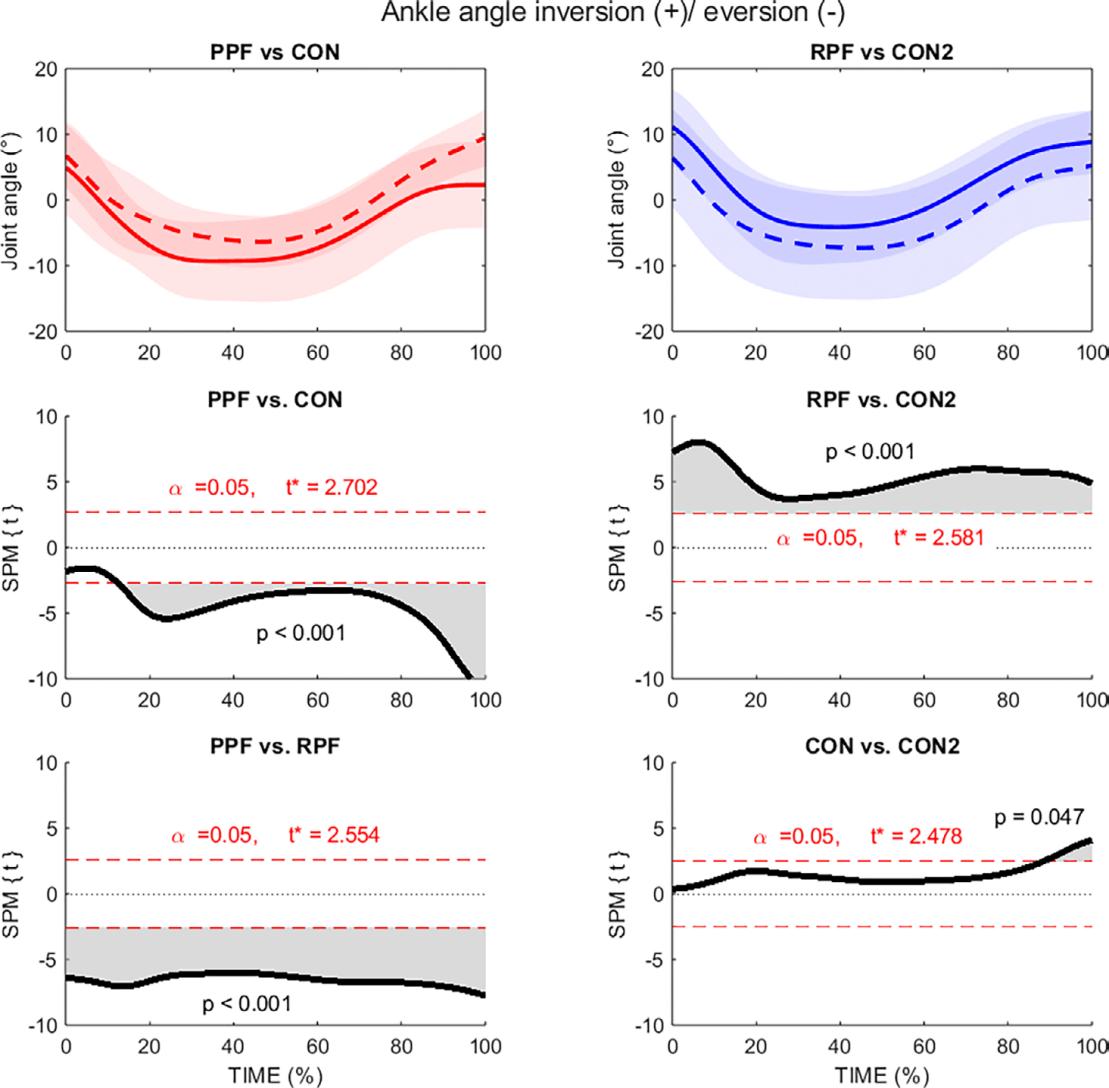
Statistical parametric mapping of ankle joint angle in the frontal plane (inversion (+)/eversion (−)) during stance phase of running. PPF, red solid line; CON1, red dashed line; RPF, blue solid line; CON2, blue dashed line.

In addition, RPF showed reduced eversion compared to CON2 across the entire stance phase (0%–100% stance, *p* < 0.001; average mean difference = 3.7°; *d* = 0.55). The largest mean differences were observed within 0%–20% of stance (average mean difference 0%–20% stance = 4.8°; average ES: *d* = 0.70) and within 50%–100% of stance (average mean difference = 3.7°; average ES: *d* = 0.55). No differences were seen between control groups up to 90% of stance (Figure 4).

**FIGURE 5:**
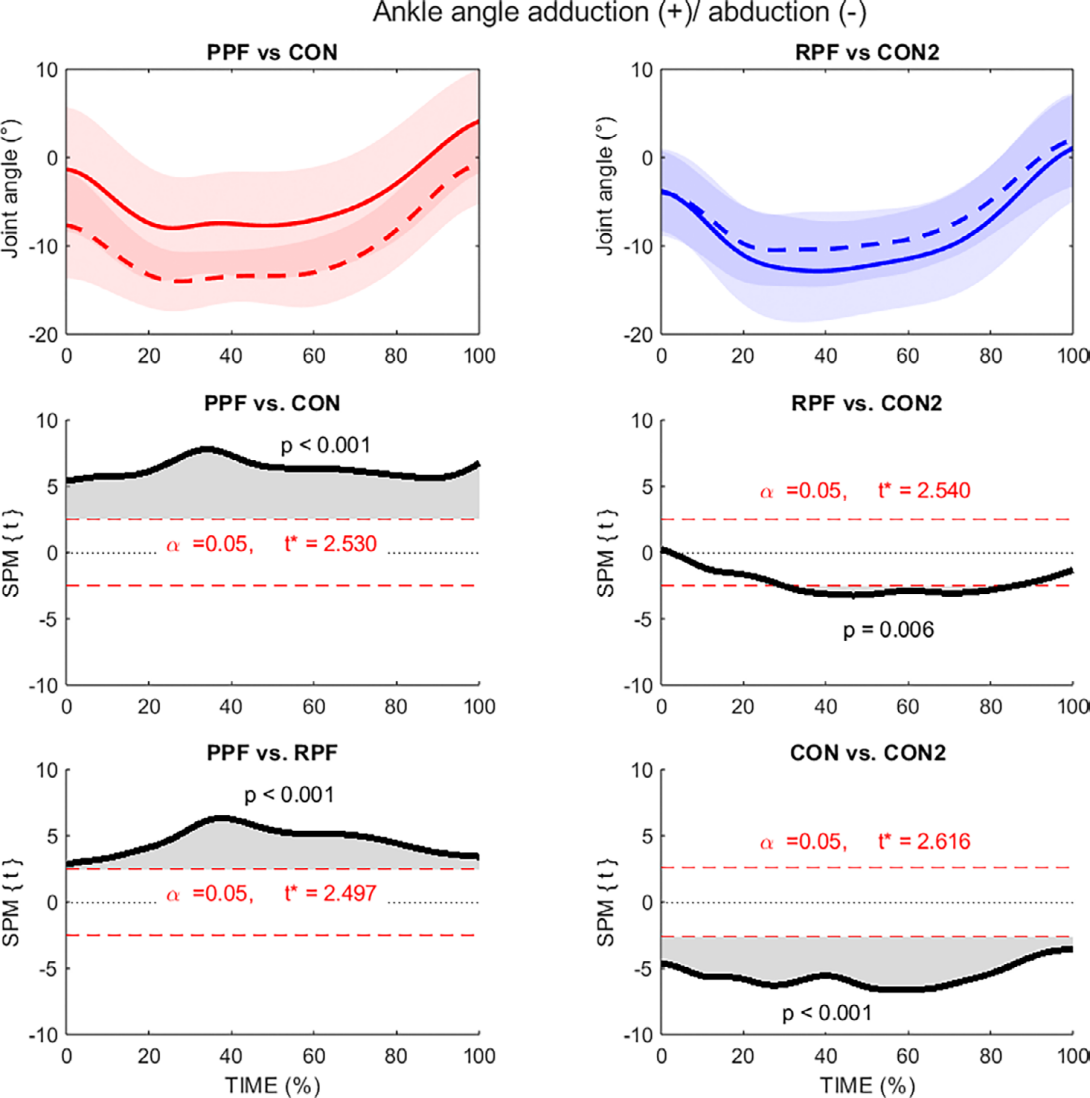
Statistical parametric mapping of ankle joint angle in the transversal plane (adduction (+)/abduction (−)) during stance phase of running. PPF, red solid line; CON1, red dashed line; RPF, blue solid line; CON2, blue dashed line.

The comparisons in the transverse plane showed interesting results (Figure 5), the CON1 and RPF groups displayed significantly greater ankle abduction angles (relative position of the foot to the shank, toward toeing out) than the PPF group during entire stance (0%–100% stance, *p* < 0.001, *d* = 1.06; *d* = 0.69). On the contrary, RPF also displayed greater abduction compared to its CON2 (30%–80% stance; *p* < 0.001; *d* = 0.40). However, there were also differences between the control groups (0%–100% stance; *p* < 0.001; *d* = 0.86).

## 4 Discussion

The aim of this study was to compare profiles of vertical ground reaction force and ankle angles during the stance phase among three groups of runners with different injury status of plantar fasciitis (resolved plantar fasciitis (RPF)), prospective cases of plantar fasciitis (PPF), and healthy controls (CON1 and CON2). First, we hypothesized that there would be no differences in the VGRF profiles among the groups. Second, we hypothesized that the PPF group would have a different pattern in the ankle angle profiles than RPF and CON1. Specifically, the PPF group would display a greater eversion angle during stance compared to CON1 and RPF. On the contrary, CON1 would exhibit a greater ankle abduction angle through the stance.

The present study identified distinct kinetic and kinematic running characteristics among individuals with a history of plantar fasciitis (RPF), those who prospectively developed the injury (PPF), and healthy controls (CON1 and CON2). In the VGRF profiles, both injured groups PPF and RPF exhibited significantly greater forces than both CON1 and CON2, with PPF and RPF most exceeding their controls notably around 30% of stance phase. This time point is of particular interest as it coincides with previously reported significant differences in eversion maxima in an earlier study [8] and is consistent with the greater eversion angle observed in PPF during this study. While higher vertical loading amplitudes have been hypothesized to increase plantar fascia strain [18, 21, 34], recent prospective findings demonstrated that vertical instantaneous loading rate (VILR) was not a risk factor for plantar fasciitis [8]. However, VILR is typically calculated using one of several methods: within the first 50 milliseconds after initial contact, from footstrike to the impact peak, between 20%–80% of the impact peak, or between initial contact and 13% of the stance phase [35, 36]. This indicates that, in the current study, the observed later differences in VGRF (with peaks between 23%–35%) more likely reflect loading mechanisms involving sustained forces in combination with altered kinematics, particularly in the frontal plane [8], rather than impact transient effects alone.

In the sagittal plane, both control groups (CON1 and CON2) exhibited a more dorsiflexed ankle at initial contact and early stance (0%–16% of the stance phase) than injured groups (PPF and RPF). From a mechanical stress perspective, there appears to be a conflict in the existing literature regarding plantar fasciitis. While Chen et al. (2019) [37] reported higher tensile forces in the plantar fascia during forefoot running, on the contrary Johnson et al. (2020) [21] discussed higher vertical instantaneous loading rates in runners with plantar fasciitis which are typically associated with rearfoot strike. Johnson et al. (2020) [21] suggested that this strike pattern results in greater rates of vertical arch deformation, thereby increasing the strain rate on the plantar fascia and potentially contributing to pain. It should also be noted that rearfoot strike patterns were more prevalent in the control groups compared to both injured groups. However, as no significant differences in strike index were observed between PPF and RPF, foot strike classification alone does not appear to account for the observed kinematic and kinetic differences.

Some treatment strategies for plantar fasciitis are based on stretching interventions [5, 38], which may influence or reflect this biomechanical adaptation. In light of the results presented in the current study, we may speculate whether resolved plantar fasciitis and continued running activity could be underpinned by adaptive changes in plantar fascia mechanical properties in RPF runners. This may be related to a more effective windlass mechanism, potentially resulting in a lower chance of re‐occurrence of the injury. Notably, RPF runners displayed greater plantar flexion during late stance (60%–90%), which may reflect altered energy storage and release within the plantar fascia–arch complex. The windlass mechanism refers to the process by which dorsiflexion of the toes during the push‐off phase of gait tensions the plantar fascia, thereby increasing the arch height and contributing to efficient force transmission during running. In addition, it was shown that runners with acute plantar fasciitis also change their foot mechanics during running leading to attenuate windlass mechanism in order to avoid greater stress and pain in plantar fascia before the toe‐off [19]. However, these sagittal‐plane differences appear to be secondary findings and should be interpreted in the broader context of the pronounced frontal‐plane alterations and elevated VGRF observed in the PPF group, which may represent the primary mechanical characteristics associated with injury status in the current study.

As hypothesized, the frontal plane analysis revealed pronounced differences in ankle eversion angle profile between groups. The PPF exhibited greater and more prolonged eversion throughout the entire stance compared with both RPF and CON1. This is consistent with elevated or prolonged pronation which is often thought a recognized risk factor for plantar fasciitis [8, 39]. The RPF group demonstrated a reduced eversion across stance and greater inversion in early stance. This may reflect a protective adaptation for limiting medial fascia strain and avoiding pain during weight acceptance, potentially shifting load distribution toward the lateral foot structures. Both CON1 and CON2 groups also exhibited a more balanced eversion profile “buffer‐zone” and avoiding the extreme values observed in the prospective injury group. Importantly, the PPF group demonstrated significantly higher VGRF than the CON1 group at the time of peak eversion, occurring at approximately 30%–40% of stance. This finding should be considered during a running assessment when selecting motion‐control/stability running shoes or using motion control insoles [39, 40, 41], particularly for individuals who exhibit excessive ankle eversion combined with elevated VGRF around 30% of stance.

In the transverse plane, the resolved RPF and control (CON1) groups demonstrated greater ankle abduction (i.e., toe‐out)—compared to prospectively injured group throughout the stance phase. However, the potential protective role of ankle abduction remains unclear [8]. One possible explanation is that the foot progression angle observed in the CON1 group may redistribute plantar loading across the medial arch. In addition, previous studies have suggested that tendons consist of physiologically active tissue capable of structural adaptation [42, 43]. Specifically, changes in collagen synthesis may enhance tissue function following targeted eccentric strength and/or stretching interventions [5]. In this context, dynamic movement in the transverse plane may stimulate collagen turnover mechanism [43]. However, this potential explanation of protective mechanisms in transversal plane remains highly speculative if we take in to the account that there were also significant differences between the CON1 and CON2. The existence of between‐control differences suggests that transversal‐plane findings should be interpreted with caution and may partly reflect inter‐group variability unrelated to injury status.

In addition to the biomechanical considerations, the MRI findings revealed that some PPF runners exhibited pathologically altered plantar fascia despite being asymptomatic at baseline, similarly to prospectively injured runners with Achilles' tendinopathy as recently reported by Jandacka et al. (2025) [44]. These findings may indicate that approximately 44% of PPF individuals could be in the prodromal stage of the injury, potentially associated with elevated levels of baseline TNF‐α, IL‐1RA and IL‐6, indicative of subclinical microdamage and the early phase of tissue repair [12, 13, 14]. This may suggest that certain PPF runners could be in an occult phase of tissue degeneration, which was difficult to detect clinically, as they did not yet experience pain and concurrently have not altered their running technique due to pain discomfort. This was possibly evident in the differences observed between RPF and PPF runners, particularly throughout the entire stance phase in the frontal and/or sagittal planes during late stance. For instance, Cook and Purdam (2009) [17] proposed that pain may occur at any stage of the tendon pathology continuum and that structural pathology and symptom presentation do not necessarily progress in parallel. The continuum model describes three stages of load‐induced tendon‐like connective tissue pathology: reactive tendinopathy, tendon dysrepair, and degenerative tendinopathy [17]. The reactive stage represents an early, potentially reversible response to overload, whereas later stages involve progressive matrix disorganization and degeneration. This dissociation between tissue morphology and pain may help explain why 44% of PPF runners (MRI grade 2–3) exhibited altered plantar fascia structure at baseline despite being asymptomatic. In contrast, 70% of runners in the RPF group demonstrated non‐pathological plantar fascia morphology at baseline. Within the framework of the continuum model, early reactive tissue changes may be reversible if mechanical loading is appropriately reduced. It is therefore possible that during their previous symptomatic episode, these individuals adjusted their mechanical loading by modifying their running pattern in response to pain, potentially facilitating structural recovery of the plantar fascia. Although this interpretation remains speculative, it is consistent with the concept that symptom‐driven load modification may influence tissue adaptation, as described in the tendon pathology continuum model [17].

### 4.1 Strength and Limitations of the Study

A key strength of this study is its prospective design, incorporating four groups of runners based on their plantar fasciitis injury status. These four groups did not differ in sex, age, anthropometric characteristics, aerobic capacity, running experience (years of running), training characteristics (weekly running distance, number of running episodes per week, total duration of running episodes per week, average duration of a single running episode, average speed during running episodes, average weekly elevation gained, footwear type, running surface, and environment), or occupational physical activity. The inclusion of a group with resolved plantar fasciitis who remained injury‐free during a 1‐year follow‐up provides a novel perspective on the biomechanics of running‐related injuries. Until now, most studies have compared running biomechanics between groups currently injured or with a history of injury and control groups. A matched observational design with two different injury‐stage groups and separate control groups methodologically provides less biased comparisons. Importantly, this study also included two control variables: MRI‐based evaluation of plantar fascia morphology and baseline blood cytokine concentrations. These provided additional context for interpreting the biomechanical findings and help to estimate the stage of injury. Furthermore, prospective cases in this study were confirmed by medical professionals, although the assessment did not include severity of plantar fasciitis and was not conducted by a single physician due to logistical constraints. The use of standardized laboratory‐neutral cushioned running footwear (standard running shoes) can be considered both a strength and a limitation of the study.

First, the main limitation of this study is the absence of medical verification for retrospective cases of plantar fasciitis, which were self‐reported by participants at baseline. Second, the study did not include a dynamic analysis of training characteristics prior to injury onset (i.e., a time‐event analysis). Third, although the PPF‐CON1 and RPF‐CON2 groups were generally well matched based on baseline data, small differences in some variables (e.g., prospectively measured physical activity, VO_2_max) were observed. However, none of these differences were statistically significant, but some of them reach a medium‐large effect size, particularly prospectively measured weekly running distance, average weekly duration of running activity, and average duration of single running episode in the PPF‐CON1 comparison (*r* = 0.45; *r* = 0.53; *d* = 0.78; Table 2). Fourth, another limitation of this study is that inflammatory cytokines were measured only once at baseline. This single time‐point assessment restricts the ability to establish temporal or causal relationships between inflammatory status and subsequent or retrospective injury development. Fifth, to our knowledge, there is currently no widely accepted standardized MRI grading system for plantar fascia pathology. Therefore, we used a structured grading approach integrating thickness, signal intensity, edema, and partial tears based on published radiologic descriptions [15, 16, 31] and clinical practice. Although the grading demonstrated excellent intra‐reader reliability, comparisons with studies using different imaging criteria should be interpreted cautiously. Lastly, baseline biomechanical data were collected under non‐fatigued conditions. Consequently, it is uncertain whether participants' movement patterns would differ under fatigue, which may influence the applicability of the findings to situations involving prolonged running episode in some runners. These limitations should be taken into account when interpreting the biomechanical findings.

## 5 Conclusions

The current study brought a new perspective into the assessment of running biomechanics and injury risk/protective/coping factors with regard to different stages of a running related‐injury. In general, differences in running biomechanics between runners with resolved plantar fasciitis (retrospectively injured), prospectively injured runners, and healthy controls may indicate that certain biomechanical patterns observed in retrospectively injured (resolved) runners could reflect adaptive mechanisms rather than inherent risk factors, as concluded by most previous biomechanical studies using groups of retrospectively injured runners and healthy controls. Specifically, this study identified elevated ankle eversion during the stance phase of running as a primary risk factor for the development/onset of plantar fasciitis and/or also adaptation to avoid the reoccurrence of the injury, suggesting that early correction of eversion may serve as an effective preventive strategy against this common running‐related injury. Furthermore, the findings suggest that adopting a combination of reduced ankle eversion and greater plantar flexion during late stance may represent viable coping mechanisms to prevent reoccurrence in runners who have previously recovered from plantar fasciitis and wish to continue engaging in this physical activity in a healthy and sustainable manner while respecting moderate/appropriate weekly running volume.

## 6 Perspective

This research showed importance of understanding the multi‐planar and temporal loading patterns that contribute to plantar fasciitis risk. Evidence suggests that sustained vertical ground reaction forces combined with elevated eversion may increase strain on the plantar fascia, even in the absence of high vertical instantaneous loading rates early after initial contact [8]. A primary preventive strategy could aim to replicate lower vertical ground forces and reduced eversion observed in healthy runners during the absorption phase (near peak eversion). Additionally, some runners appear to adopt compensatory patterns, such as reduced eversion and increased plantar flexion before take‐off, potentially improving windlass mechanism function. These findings highlight the need to consider not only load magnitude but also its timing and multi‐directional components in risk assessment and rehabilitation. Interventions may include pronation‐control footwear, insoles and targeted stretching. Future studies should employ time‐to‐event designs enabling survival analysis to better capture behavioral patterns (e.g., training characteristics) preceding injury onset. Moreover, research should explore ankle biomechanics in relation to plantar fasciitis and Achilles' tendinopathy, given their mechanical connection. Finally, randomized controlled trials comparing different running techniques and various footwear types could provide valuable insights for injury prevention and treatment strategies.

## Author Contributions

Design of the 4HAIE study was created by: Daniel Jandacka, Joseph Hamill, Julia Freedman Silvernail, David Zahradnik, Lukas Cipryan. Design of this study: Jan Plesek, Joseph Hamill, Daniel Jandacka. Methods: Jan Plesek, Joseph Hamill, Pavel Brtva, Daniel Jandacka. Data analyses and interpretation: Jan Plesek, Pavel Brtva, Jiri Skypala, Jan Urbaczka, Milos Golian, Daniel Jandacka, Lukas Cipryan, Jan Sustek. Writing – original draft: Jan Plesek. Writing – critical revision and editing: Jan Plesek, Joseph Hamill, Pavel Brtva, Jiri Skypala, Jan Urbaczka, David Zahradnik, Julia Freedman Silvernail, Lukas Cipryan, Milos Golian, Jan Sustek, Daniel Jandacka. All authors approved the final version of the manuscript.

## Funding

This work was supported by LERCO (CZ.10.03.01/00/22_003/0000003). Research of Excellence on Digital Technologies and Wellbeing, (CZ.02.01.01/00/22_008/0004583).

## Ethics Statement

The 4HAIE study was approved by the Ethics and Research Committee of the University of Ostrava (OU‐87674190‐2018) and was conducted in accordance with the principles of the Declaration of Helsinki. All participants signed an informed consent before the data collection.

## Conflicts of Interest

The authors declare no conflicts of interest.

## Supporting information


**Figure S1:** MRI assessment (representative grading images): (A) Grade 0 – normal thin hypointense fascia without edema; (B) Grade 1 – thickened fascia with preserved low signal (both representing non‐pathologic plantar fascia, i.e., normal to mildly altered tissue); (C) Grade 2 – thickened fascia with intermediate intrafascial signal; (D) Grade 3 – thickened fascia with heterogeneous hyperintensity and perifascial edema (both representing pathologic plantar fascia, i.e., moderately to severely altered tissue).

**Table S1:** Numbers of missing data in the pair comparisons.

## Data Availability

The data that support the findings of this study are available from the corresponding author upon reasonable request.
