# Supplementary figures and images for: Ankle Joint Biomechanics in Recreational Runners with Resolved and Incident Plantar Fasciitis: A One‐Year Prospective 4HAIE Cohort Study
Source: Scand J Med Sci Sports. 2026 Apr 13;36(4):e70281. doi: 10.1111/sms.70281 (PMC13077024; doi:10.1111/sms.70281)

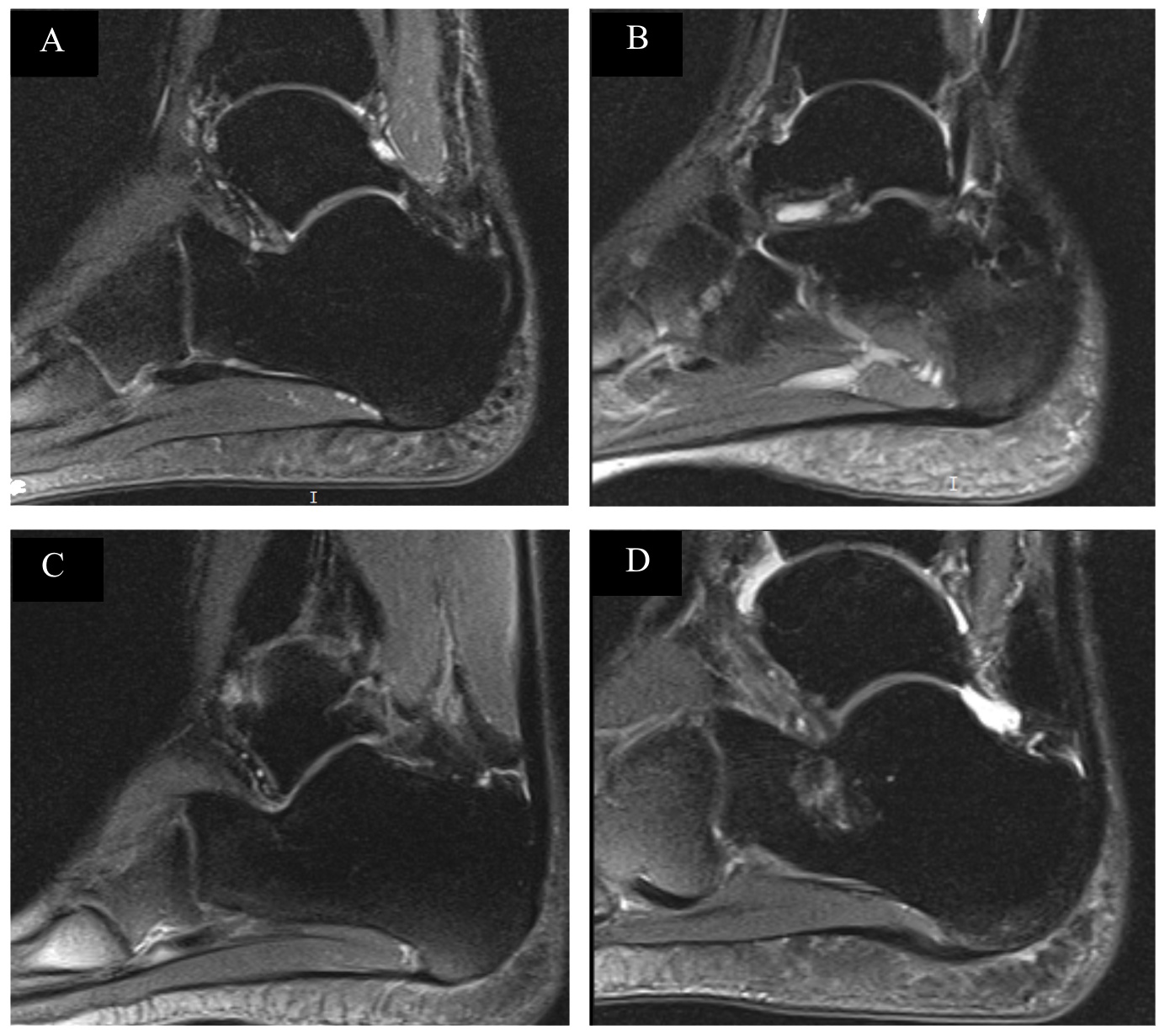

Supplement: Supplementary file 1 — Figure S1: MRI assessment (representative grading images): (A) Grade 0 – normal thin hypointense fascia without edema; (B) Grade 1 – thickened fascia with preserved low signal (both representing non‐pathologic plantar fascia, i.e., normal to mildly altered tissue); (C) Grade 2 – thickened fascia with intermediate intrafascial signal; (D) Grade 3 – thickened fascia with heterogeneous hyperintensity and perifascial edema (both representing pathologic plantar fascia, i.e., moderately to severely altered tissue). [file SMS-36-e70281-s002.tiff]
