# Supplementary material for: Ankle Joint Biomechanics in Recreational Runners with Resolved and Incident Plantar Fasciitis: A One‐Year Prospective 4HAIE Cohort Study
Source: Scand J Med Sci Sports. 2026 Apr 13;36(4):e70281. doi: 10.1111/sms.70281 (PMC13077024; doi:10.1111/sms.70281)
Supplement: Supplementary file 2 — Table S1: Numbers of missing data in the pair comparisons. [file SMS-36-e70281-s001.docx]

**Supplemental Table 1 – Numbers of missing data in the pair comparisons.**

|  | **Prospective plantar fasciitis**  **(PPF)** | **Controls**  **(CON1)** | **Resolved plantar fasciitis**  **(RPF)** | **Controls 2**  **(CON2)** |
| --- | --- | --- | --- | --- |
| N | 10 (100%) | 10 (100%) | 14 (100%) | 14 (100%) |
| Sex (F/M; N (%)) | 0 (0%) | 0 (0%) | 0 (0%) | 0 (0%) |
| Age (year) | 0 (0%) | 0 (0%) | 0 (0%) | 0 (0%) |
| Running distance – retrospective questionnaire (km/week) | 0 (0%) | 0 (0%) | 0 (0%) | 0 (0%) |
| BMI | 0 (0%) | 0 (0%) | 0 (0%) | 0 (0%) |
| Height (cm) | 0 (0%) | 0 (0%) | 0 (0%) | 0 (0%) |
| Mass (kg) | 0 (0%) | 0 (0%) | 0 (0%) | 0 (0%) |
| Body Fat (%) | **1 (10%)** | 0 (0%) | 0 (0%) | **1 (7%)** |
| Average running distance per week (km/week) | **1 (10%)** | 0 (0%) | 0 (0%) | **1 (7%)** |
| Average duration of running activity per week (min/week) | **1 (10%)** | 0 (0%) | 0 (0%) | **1 (7%)** |
| Average number of running episodes per week (N/week) | **1 (10%)** | 0 (0%) | 0 (0%) | **1 (7%)** |
| Average duration of one running episode (min) | **1 (10%)** | 0 (0%) | 0 (0%) | **1 (7%)** |
| Average running speed in running episodes (km/h) | **1 (10%)** | 0 (0%) | 0 (0%) | **1 (7%)** |
| Average elevation gained per week (m/week) | **1 (10%)** | 0 (0%) | 0 (0%) | **1 (7%)** |
| VO2Max | 0 (0%) | 0 (0%) | 0 (0%) | 0 (0%) |
| Strike index | 0 (0%) | 0 (0%) | 0 (0%) | **1 (7%)** |
| Cadence | 0 (0%) | 0 (0%) | 1 (7%) | 0 (0%) |
| MRI | **1 (10%)** | 0 (0%) | 0 (0%) | **1 (7%)** |
| IL-1 | **2 (20%)** | 0 (0%) | 0 (0%) | 0 (0%) |
| IL-1ra | **2 (20%)** | 0 (0%) | 0 (0%) | 0 (0%) |
| IL-6 | **2 (20%)** | 0 (0%) | 0 (0%) | 0 (0%) |
| IL-10 | **2 (20%)** | 0 (0%) | 0 (0%) | 0 (0%) |
| TNF | **2 (20%)** | 0 (0%) | 0 (0%) | 0 (0%) |
| CRP | **1 (10%)** | 0 (0%) | 0 (0%) | 0 (0%) |
| VGRF | 0 (0%) | 0 (0%) | 0 (0%) | 0 (0%) |
| Ankle angle - X | 0 (0%) | 0 (0%) | 0 (0%) | 0 (0%) |
| Ankle angle - Y | 0 (0%) | 0 (0%) | 0 (0%) | 0 (0%) |
| Ankle angle - Z | 0 (0%) | 0 (0%) | 0 (0%) | 0 (0%) |
|  |  |  |  |  |
| Total number of trials used for paired t-test (N) | 80 (100%) | 80 (100%) | 112 (100%) | 112 (100%) |
| VGRF | 0 (0%) | 0 (0%) | 4 (4%) | 4 (4%) |
| Ankle angle - X | 0 (0%) | 0 (0%) | 10 (9%) | 10 (9%) |
| Ankle angle - Y | 0 (0%) | 0 (0%) | 10 (9%) | 10 (9%) |
| Ankle angle - Z | 0 (0%) | 0 (0%) | 10 (9%) | 10 (9%) |
